# Minimally invasive prostatic urethral lift (PUL) efficacious in TURP candidates: a multicenter German evaluation after 2 years

**DOI:** 10.1007/s00345-018-2494-1

**Published:** 2018-10-03

**Authors:** Karl-Dietrich Sievert, Martin Schonthaler, Richard Berges, Patricia Toomey, Desiree Drager, Annika Herlemann, Florian Miller, Ulrich Wetterauer, Bjorn Volkmer, Christian Gratzke, Bastian Amend

**Affiliations:** 1 Department of Urology, University Clinic of Tubingen, Tuebingen, Germany; 2 Department of Urology, Medical University of Vienna, Vienna, Austria; 3 Department of Urology, University Clinic of Rostock, Rostock, Germany; 4 Department of Urology, University Clinic of Freiburg, Freiburg, Germany; 5 Department of Urology, PAN Klinik Köln, Cologne, Germany; 6 Department of Urology, Klinikum Kassel, Kassel, Germany; 7 Department of Urology, University Clinic of Munich, Munich, Germany; 8 Klinikum Lippe Detmold, Detmold, Germany

**Keywords:** Prostatic urethral lift (PUL), Benign prostatic hyperplasia (BPH), Transurethral resection of prostate, Minimally invasive surgical therapy, LUTS

## Abstract

**Introduction:**

Successful outcomes have been reported for the treatment of lower urinary tract symptoms (LUTS) with the prostatic urethral lift (PUL) in a number of clinical investigations. Our aim was to investigate PUL outcomes in patients treated in a day-to-day clinical setting without the rigid exclusion criteria of clinical studies.

**Materials and methods:**

We investigated the outcome of the PUL procedure at five German departments during the initial period when PUL was approved for the clinic (10/2012–06/2014). All candidates for transurethral resection of the prostate (TURP) received PUL information and were given the choice of procedures. The only exclusion criterion was an obstructive median lobe. No patients were excluded because of high post-void residual volume (PVR), prostate size, retention history or LUTS oral therapy. Maximum urinary flow (Qmax), PVR, International Prostate Symptom Score (IPSS) and Quality of Life (QOL) were assessed at baseline, 1, 6, 12, and 24 months after surgery.

**Results:**

Of 212 TURP candidates, 86 choose PUL. A mean of 3.8 (2–7) UroLift implants were implanted in patients of 38–85 years with a prostate size of 17–111 ml over 57 (42–90) min under general or local anesthesia. Thirty-eight (38.4%) patients had severe BPH obstruction and would have been denied PUL utilizing previously reported study criteria. Within 1 month 74 (86%) reported substantial symptom relief with significant improvements in Qmax, PVR, IPSS, and QOL (*p* < 0.001) that was maintained within the follow-up. Sexual function including ejaculation was unchanged or improved. No Clavien–Dindo Grad ≥ 2 was reported postoperatively. Eleven (12.8%) patients were retreated over 2 years. Twelve (86%) of 14 patients presenting with chronic urinary retention were catheter free at last follow-up.

**Conclusion:**

PUL is a promising surgical technique that may alleviate LUTS, even in patients with severe obstruction.

## Introduction

Benign prostatic obstruction (BPO), as an effect of benign prostate hyperplasia (BPH), is very common in men over the age of 50 and can result in bothersome lower urinary tract symptoms (LUTS) [1, 2]. Pharmacotherapy has been reported to achieve improvements of 30–40% in symptoms with reductions in the International Prostate Symptom Score (IPSS) and increases of 20–25% in urinary flow (Qmax), especially in patients with a smaller prostate (< 40 ml) [1–4]. However, in general practice, clinicians often find that the drug-related dropout rates can be high because of insufficient response, side effects or lifetime commitment necessity [5, 6].

For patients with moderate-to-severe LUTS who do not wish to take drugs and/or are sufficiently bothered by LUTS symptoms, a number of surgical treatment options may be considered [1, 2, 7]. Transurethral resection of the prostate (TURP) remains the primary treatment option with significant long-term improvement in clinical outcomes and low retreatment rates 10 years after intervention, attesting to its long-lasting effects [1, 2]. While TURP achieves maximal effect on urinary flow, relief can come with a very significant side-effect profile [8, 9]. Reported perioperative and long-term complications include bleeding (requiring blood transfusions), acute urinary retention, clot retention, urinary tract infection, erectile dysfunction, retrograde ejaculation, bladder neck or urethral stricture [1, 2]. Because of these complications, new therapeutic treatment options using innovative technologies to relieve patient symptoms are rapidly emerging [10–13].

The prostatic urethral lift (PUL) procedure is a novel, minimally invasive therapeutic option for the treatment of LUTS that can be performed without tissue removal or involvement of the inner sphincter [6, 14]. A solid literature base demonstrates that the PUL procedure results in rapid and significant improvement in voiding and symptom scores. These published results are superior to oral therapy and do not involve the removal or destruction of prostatic tissue, significant perioperative morbidity issues or the long-term complications of ablative surgery [15–18]. Although PUL has proven to achieve significant relief of LUTS in men with moderate-to-severe symptoms, controlled clinical studies have focused exclusively on men with higher residual volume or previous urinary retention. It is understandable that to prove the validity and safety of a new procedure, randomized clinical trials in a rigorously selected population of subjects are necessary [19]. While recruiting patients for the BPH6 clinical study, we hypothesized that the less invasive PUL could be a successful substitute for TURP, even if BPO was severe. We, therefore, investigated the outcome of PUL in an unselected population of BPO patients who had already been deemed to be candidates for TURP.

## Materials and methods

### Study design and patients

We investigated the outcome of the newly approved PUL procedure in men treated for LUTS during the period from 10/2012 through 06/2014 at five German urology departments. Data were retrieved from a prospectively designed database. Patients with confirmed moderate-to-severe BPO-related LUTS, that were unresponsive to oral therapy, were considered eligible for PUL if they were candidates for surgical ablation with TURP. All patients considered for TURP had undergone a cystoscopy and transrectal digital and ultrasound examination. The only exclusion criterion for PUL was an obstructive median lobe seen during the initial cystoscopy. No patients were refused PUL because of prostate size, high post-void residual (PVR) or history of retention. Eligible patients received information about both procedures, including the relative risks and benefits of PUL and TURP, and were given the option of the PUL procedure as a substitute for TURP. All patients fulfilled the German health care and legal system requirements and provided written informed consent for the procedure.

### Surgical procedure

In the PUL procedure, a transurethrally inserted delivery device deploys small, custom-sized implants (UroLift^®^, NeoTract, Inc., Pleasanton, California) through the prostate in order to separate the prostatic lateral lobes, thereby holding open the voiding channel. Detailed instructions for implanting the devices have been described in earlier publications [14, 15, 20, 21]. The procedure was performed in either general or local anesthesia [15]. The choice of the type of anesthesia was given to the patient or decided based on the surgeon’s preference. For the procedure under local anesthesia, 4 °C of cold lidocain 10% (50 ml) was given through a transurethral that was followed by an injection of 4 °C cold lidocain gel (10 ml) into the urethra. A penile clamp was placed until the PUL procedure was performed (30–45 min). In case the patient would not tolerate the procedure under local anesthesia, an intravenous catheter was placed to provide the patient with either additional pain medication or, if necessary, the switch to full anesthesia.

### Assessments

Follow-up visits were scheduled at 1, 6, 12, and 24 months postoperatively. Maximum urinary flow (Qmax), PVR, and IPSS (range 0–35; 0 = no symptoms), including single related quality of life question (QOL) (score: range: 0–6; 6 = “terrible”), were assessed at baseline and at each follow-up visit to evaluate PUL effectiveness [22]. During these visits, all patients were questioned whether they had experienced any changes in sexual function from their baseline reports.

### Statistical analysis

Each department with descriptive statistics summarized patient characteristics and outcomes. JMP (JMP 11.1.1, SAS Institute Inc., Cary, USA) was used for statistical analysis. Data have been analyzed for normality using the Shapiro–Wilk test. The Student *t* test was used in parametric distributed data and the Wilcoxon/Kruskal–Wallis test (rank sum test) was used for non-parametric data. A *p* value of < 0.05 was considered as a significant difference (two-sided tests).

## Results

### Patient and operative characteristics

Of 212 patients eligible for TURP, 138 (65%) patients were also eligible for the PUL procedure; 74 patients were excluded because of a prominent median lobe or a prominent bladder neck. 86 (62%) of the 138 patients who met eligibility criteria for PUL chose to undergo the procedure. The remaining patients elected to undergo TURP as discussed with their referring urologist.

At baseline (Table 2), the mean Qmax was 11.24 ml/s (standard deviation [22] = 3.16 ml/s; range 4–19 ml/s), the mean PVR was 149.53 ml (SD = 251.46 ml; range 10–1600 ml), the mean IPSS was 20.82 (SD 6.52; range 5–34), and the mean QoL score was 4.14 (SD 1.22; range 1–6).

Fourteen patients (16.3%) had a history of an indwelling urinary catheter (1–2 months, with a previous unsuccessful attempt of catheter removal). Seven patients (8.1%) had a PVR value greater than 250 ml, including three patients in the range of > 250–350 ml, two patients between 500 and 600 ml, and two patients 1500–1600 ml, indicating virtual urinary retention at the time of implantation. Twelve patients (14%) had prostate volumes ≥ 60 ml, including 5 with volumes ≥ 80 ml. Thus, a total of 33 patients (38.4%) had severe BPO as manifested by a history of urinary retention, high PVR or an enlarged prostate gland. Coincidentally, 33 patients (38.4%) were taking α-blockers at the time of implantation. Of those under α-blocker, 11 (39%) complained of ejaculatory problems related to the medication.

### Surgical procedure

The mean operative time was 57 min (Table 1). No intraoperative adverse events were recorded. All patients undergoing PUL received an in-dwelling urinary catheter as required under German insurance, which was removed in 5 h to 4 days (mean 29.5 h). The mean postoperative hospitalization time was in average two (0–12) days. Two patients required prolonged observation due to non-urological comorbidities. The number of implants varied between 2 and 7, depending on the prostate size (Table 1). General anesthesia was used in 64 patients and local anesthesia in 24. Of the 24, about 80% were performed in one clinic center that performed 51% of the cases in local anesthesia. For those patients who underwent local anesthesia, no further pain medication or full anesthesia was required.

**Table 1:** Patient characteristics

Characteristic	*N* = 86 (%)
Age (year)	
Mean (SD)	66.2 (11.5)
Range	38–85
Prostate volume (ml)	
Mean (SD)	43 (18.8)
Range	17–111
Operative time (min)	
Mean (SD)	57 (12)
Range	42–90
Number of implants per patient	
Mean (SD)	3.8 (1.4)
Range	2–7
Previous catheterization, *n* (%)	14 (16.3)
Pharmaceutical treatment of LUTS, *n*	33 (38.4)

*SD* standard deviation, *LUTS* lower urinary tract symptoms

### Outcomes and adverse events

**Fig. 1:**
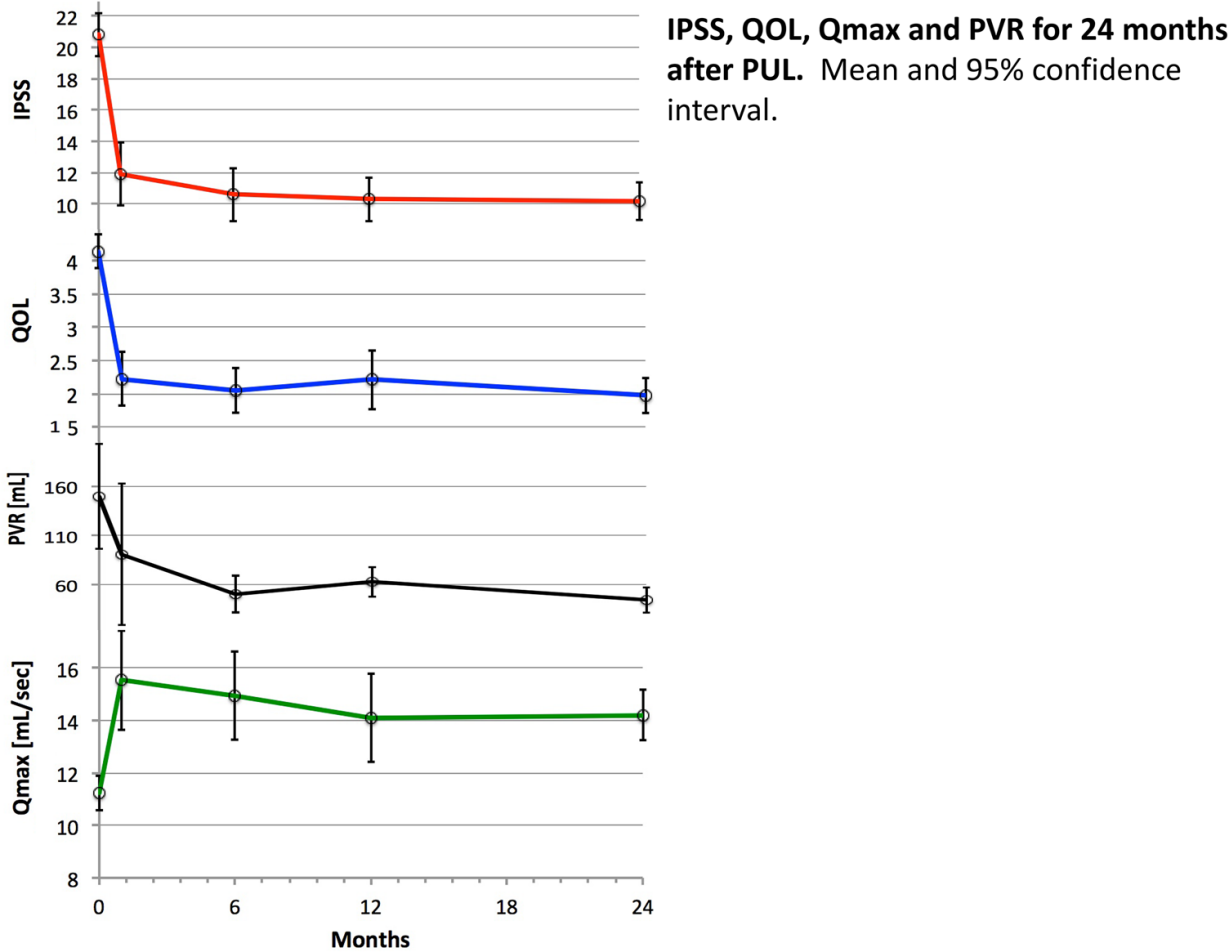
IPSS, QOL, Qmax and PVR for 24 months after PUL. Mean and 95% confidence interval

Of the 86 patients, 74 (86%) reported substantial symptom relief within 1 month, with significant decreases in mean IPSS and QOL scores, which were maintained throughout the follow-up period (Fig. 1, Table 2). Significant functional improvement was also seen, with a decrease in mean PVR from 150 ml at baseline to 51 ml (6 months) and 45 ml (24 months), and an increase in mean Qmax from 11.1 ml/s to a peak at 1 month of 15.5 ml/s and leveling to 14.2 ml/s at 24 months. Those patients with an preoperative in-dwelling catheter and were able to void postoperative with an acceptable PVR (less than 1/3 of the bladder capacity) had a Qmax of 12.8 ml/s (6–19.6 ml/s) and a PVR of mean 43.3 ml (0–80 ml) at 6 months postoperative, which remained unchanged on this level. Improvements in symptoms, flow and quality of life were maintained throughout the 24-month observation period, and the changes from baseline were significant at each interval up to 1 year (Fig. 1, Table 2). The sub-analysis of this group verified that the outcome became almost the same after 12 months and a similar effect was seen in those patients with a more severe obstruction (Table 3).

**Table 2:** IPSS, QOL, PVR, and Qmax at baseline, 1, 6, 12 and 24 months for patients after PUL

	Baseline	1 month	6 months	12 months	24 months
IPSS					
*N*	86	48	41	42	41
Mean ± SD	20.82 ± 6.52	11.92 ± 7.13	10.59 ± 5.61	10.29 ± 4.70	10.17 ± 3.93
95% CI	19.44–22.20	9.90–13.94	8.87–12.31	8.87–11.71	8.97–11.37
*p* value	–	< 0.0001	< 0.0001	< 0.0001	< 0.0001
QoL					
* N*	86	45	42	38	44
Mean ± SD	4.14 ± 1.22	2.22 ± 1.38	2.05 ± 1.10	2.21 ± 1.38	1.98 ± 0.90
95% CI	3.88–4.40	1.82–2.62	1.72–2.38	1.77–2.65	1.71–2.25
*p* value	–	< 0.0001	< 0.0001	< 0.0001	< 0.0001
PVR (ml)					
*N*	86	41	41	33	41
Mean ± SD	149.53 ± 251.46	91.05 ± 235.06	50.85 ± 61.12	62.97 ± 44.22	44.63 ± 42.27
95% CI	96.33–202.68	19.10–163.00	32.14–69.56	47.88–78.06	31.69–57.57
*p* value	–	0.2951	< 0.01	< 0.005	< 0.01
Qmax (ml/s)					
*N*	86	42	46	34	43
Mean ± SD	11.24 ± 3.16	15.54 ± 6.27	14.95 ± 5.82	14.11 ± 5.02	14.21 ± 3.28
95% CI	10.57–11.91	13.64–17.44	13.27–16.63	12.42–15.80	13.23–15.19
*p* value	–	0.005	< 0.001	< 0.001	0.005

**Table 3:** Subgroup of those patients who initially had either a high post-void residual (> 250 ml, > 50% of their bladder capacity or an indwelling catheter) and/or a prostate volume > 60 ml

	Baseline	1 month	6 months	12 months	24 months
IPSS					
*N*	33	24	23	23	22
Mean ± SD	19.83 ± 6.38	9.58 ± 6.13	9.6 ± 3.33	9.82 ± 1.66	11.17 ± 3.59
95% CI	17.14–22.53	5.69–13.48	7.75–11.45	8.70–10.94	8.89–13.45
*p* value	–	< 0.0001	< 0.0001	< 0.0001	0.0004
QoL					
*N*	33	25	23	17	25
Mean ± SD	3.79 ± 1.35	1.82 ± 0.98	2.00 ± 0.96	2.00 ± 0.85	1.93 ± 0.83
95% CI	3.22–4.36	1.16–2.48	1.45–2.55	1.46–2.54	1.45–2.41
*p* value	–	0.0003	< 0.0001	0.0002	0.0004
PVR (ml)					
*N*	31	19	20	15	21
Mean ± SD	333.71 ± 429.59	202.75 ± 419.77	74.62 ± 92.95	75.11 ± 57.71	63.33 ± 54.66
95% CI	138.17–29.26	63.96–469.46	18.45–30.78	30.75–119.47	28.60–98.06
*p* value	–	0.0149	0.0009	0.0377	0.0174
Qmax (ml/s)					
*N*	32	12	15	10	14
Mean ± SD	9.53 ± 2.61	14.51 ± 6.11	14.23 ± 3.61	12.45 ± 3.50	13.42 ± 2.97
95% CI	8.41–10.66	10.62–18.39	12.23–6.23	9.95–14.95	11.70–15.14
*p* value	–	0.0004	0.0003	0.0438	0.0031

Eleven patients (12.8%) reported persistence of LUTS or had remaining increased PVR, and retreatment was necessary. Cystoscopic evaluation indicated insufficient prostatic de-obstruction in two patients due to deficient implant positioning. Those patients were offered two alternatives: TURP or additional implants. One patient elected a second PUL procedure and experienced satisfactory resolution of LUTS with clinically significant lasting improvement of Qmax, PVR, IPSS, and QoL, whereas the other patient did not show meaningful improvement and declined any further treatment. The other nine patients underwent TURP. Eight of the nine experienced symptom resolution without complications or sequela from their in-dwelling prostatic implants, but one patient (whose initial PVR was 1600 ml) remained with a significant PVR after TURP and, therefore, continued with a suprapubic catheter.

Because after 2 years 45 patients (53%) did not return for the follow-up, the referring urologists were contacted. They were asked the following questions: (a) has the patient been seen since the procedure and (b) if the patient has been seen within the last half year and, if so, have any further surgical treatments been performed or additional/new oral medication been prescribed. The information in the patient charts revealed: all patients were seen after the surgery. At 2 years, 12 patients had not visited their urologist within the last 6 months. Of the remaining 33 patients, 28 had been seen within the last 6 months and none had underwent any additional surgical treatment or received any related oral drug treatment. Five patients did not want any further surgical treatment despite suggestions by the referring urologist.

Postoperatively, 12 (14.0%) patients experienced transient dysuria and hematuria secondary to the rigid cystoscopy/procedure, and 3 (3.5%) patients reported vague pelvic pain for less than a month. With regard to the Clavien–Dindo grading system, none of the patients recorded more than a Grade 2. Sexual function including ejaculation was unchanged or even improved with those who reported sexual activity prior to surgery. Of 11 patients reporting ejaculatory dysfunction at baseline, 3 (27.3%) patients reported improved ejaculatory function after PUL. By month one 57 (66.3%) patients discontinued all LUTS medication.

## Discussion

### Patient group

In the German healthcare system, patients are commonly referred to urologic surgeons typically after failed attempts with pharmaceutical treatments and often with late stage urodynamic issues due to years of insufficient treatment of LUTS. While PUL has not been studied in this specific population, these patients were nonetheless often attracted to the less invasive nature of this treatment, when compared to TURP or other ablative procedures. For this reason, we sought data outside of rigid study protocols and was reflective of our daily clinical practices. We offered PUL as an alternative procedure to all patients who were candidates for TURP, without restrictions as to severity of prostatic obstruction. Because PUL was designed for patients with lateral lobe obstruction, we excluded only patients with an obstructive median lobe seen on the initial cystoscopy. Previous trials also had many other exclusion criteria, such as oral pharmacotherapy without washout, history of urinary retention, decompensated urinary bladder, PVR greater than 250 ml, recurrent prostate-related hematuria, and prostate volume greater than 60 ml [21–24]. A limitation of this study may be that those patients who received PUL were not compared against one of the accepted surgical treatments. However, in parallel, the formal BPH-6 study, which did comparisons with TURP, was also enrolling patients [19] so we were able to compare our results with those of the randomized study.

### Comparison with other PUL studies

Of the patients enrolled, 38% suffered from severe obstruction that would have excluded them from prior clinical studies. Despite inclusion of these patients, our results are comparable to those of previously published studies on PUL in rigorously selected subject populations, including the pivotal LIFT study that resulted in US Food and Drug Administration (FDA) approval of the UroLift^®^ implant system [21–24]. Symptomatic and quality of life improvements were very similar to those reported for LIFT, with 1 year IPSS and QOL improvements of 10.5 vs. 10.8 and 1.93 vs. 2.4, respectively [22]. When further examining urinary flow and residual volumes, however, a greater difference can be seen. At 1 year, Qmax increased only 2.97 versus 4.0 ml/s in LIFT, but PVR results significantly decreased 63 ml, while no significant difference was seen in LIFT [22]. These differences might be explained by the fact that our study included men with much lower baseline flow rates and much higher residual volume or even those with an in-dwelling catheter. Very low flow rates may be a function of detrusor functionality, where dis-obstruction may have a less marked effect. The additional analysis in Table 3 substantiates that the subgroup patients who would not have been accepted in a published study, demonstrated a similar outcome. Several studies have shown that PVR change is typically not significant and is poorly reproducible in populations where high PVR is excluded [25, 26].

Adverse effects were minimal and the rate appeared to be even lower than that in the LIFT clinical study [22]. Perhaps this could be due to the fact that a third of our patients continued α-blockers therapy for a month following the implant. Also, the LIFT study involved a follow-up at 14 days, where specific questions were asked to elicit adverse event details, while our first follow-up was at 1 month with typical general history questions asked.

Our implantation execution differed from the LIFT experience [22]. We used general anesthesia in 62 of the 86 patients (72%), a postoperative indwelling catheter for 1.22 days, and 2.17 days of hospitalization. In the LIFT study and other studies conducted in North America, patients under local anesthesia usually were not catheterized and did not require hospitalization. With increasing surgeon experience, our operative times decreased, and 24 of our patients underwent the procedure successfully under local anesthesia. Our different delivery of care was not related to our inclusion of more severely obstructed patients. The difference was largely related to the dynamics of the German health care reimbursement system that is different to the United States.

Our results demonstrated durability of the improvement in voiding up to 24 months as judged by symptom score, PVR, and Qmax, corroborating the previous PUL studies, even in the group of those who would have been excluded in any previous study (Table 3). Reviewing the patient charts verified that those who did not return for the follow-up investigation had a similar outcome. Our 12.8% retreatment rate at 2 years is reasonable and within range of prior reports though somewhat higher than the 7.5% retreatment rate reported for LIFT at 2 years [22, 27]. Of the 11 men requiring retreatment, 5 (45%) were within the 33 classified as severely obstructed 2 with baseline retention and 3 with PVR > 300 ml). A potential important take away from the retreated patients (2 PUL; 9 TURP) may be that, with PUL, “bridges are not burned”, because the PUL procedure does not preclude future BPH therapy, including TURP or further retreatment with PUL. All follow-up retreatments were conducted without incident or complication.

As might be expected when treating men with severe obstruction, one patient did not respond to either PUL or even later with TURP and remains on a suprapubic catheter due to detrusor decompensation. In a larger, less severely obstructed cohort, Roehrborn demonstrated PUL durability of 86% at 4 years [27]. Sonksen et al. reported that, while 1-year retreatment for PUL and TURP were 7 and 6%, respectively, if treatment for complications were included, overall re-intervention for TURP rose to 14% [24].

Preservation of sexual function observed in these patients was consistent with previous studies focusing on possible sexual side effects following PUL [24, 28]. Our results indicated no procedure-related impotence or onset of ejaculatory dysfunction. In fact, in our evaluation, 3 of 11 patients (27%) with preoperative ejaculatory dysfunction reported improvement following PUL.

### Late-stage LUTS

Prior clinical studies have demonstrated that PUL is an efficacious treatment for men with moderate to severe symptoms (IPSS > 12), suppressed urinary flow (Qmax ≤ 12 or 15 ml/s) and low residual volume (PVR ≤ 250 ml). [21–24, 28]. This investigation shows that PUL may also be appropriate for men with later stage prostatic obstruction, as defined by urinary retention, elevated PVR or larger prostate. Fourteen patients presented with in-dwelling urinary catheterization (1–3 months with unsuccessful trial to void). Twelve (86%) men in urinary retention were successfully weaned from their catheters and remained catheter-free at the last follow-up. One of the two treatment failures also failed to void after follow-up with TURP, resulting in chronic suprapubic catheterization. Three of the seven patients with high PVR at baseline underwent TURP. Our initial experience suggests that PUL should be reserved for patients with functioning bladders. If a patient presents with very high PVR, we would recommend a period of catheterization to stabilize his bladder function and an urodynamic evaluation.

### Comparison with TURP and LASER

The BPH6 randomized study compared PUL and TURP showed that TURP offers modestly greater IPSS improvement and much greater Qmax improvement, but interestingly, there was no significant difference regarding improved quality of life [24]. While BPH6 study was of a modest size, the results for TURP mirrored those of the recent larger study of TURP and LASER [7]. In the current study, the Qmax rose modestly to a mean of 14.4 ml/s. Berges and Oelke showed in a study of 1763 German men that the Qmax represents a normal flow for this age group; thus, PUL does not give the flow correlated to TURP, but may simply improve flow characteristics to patients satisfaction [29]. As this modest Qmax improvement was associated with 51, 52, and 70% improvements in IPSS, QOL, and PVR, it may be that larger Qmax increases are not necessary for all patients to achieve an acceptable result.

Because this investigation is not a randomized prospective study comparing the extended inclusion criteria, it may be considered a limitation of the study. However, by comparing this investigation to the available surgical treatment options that are currently available, we have to keep in mind that those options are in a constant state of flux.

## Conclusions

This is the first report of the outcome of PUL when offered to all patients considering TURP, without restrictions such as a history of urinary retention, prostate size, volume, or concomitant medical treatment of LUTS. The PUL procedure yielded satisfactory results that were consistent with previously published tightly controlled study populations. Eighty-six percent of men presenting with retention were freed from catheterization, but two with very high post residual volume were not; however, this may be related to a hypocontractile detrusor muscle. For men presenting with high residual volume, urodynamic characterization of detrusor contractility and/or preceding PUL with a period of catheterized drainage may be advisable. This data could encourage clinicians to consider offering PUL therapy to a broader range of TURP candidates.

## References

[1] McVary KT, Roehrborn CG, Avins AL, Barry MJ, Bruskewitz RC, Donnell RF, Foster HE, Gonzalez CM, Kaplan SA, Penson DF, Ulchaker JC, Wei JT (2010). American urological association guideline: management of benign prostatic hyperplasia (BPH). J Urol.

[2] Gratzke C, Bachmann A, Descazeaud A, Drake MJ, Madersbacher S, Mamoulakis C, Oelke M, Tikkinen KAO, Gravas S (2016). EAU guidelines on management of non-neurogenic male lower urinary tract symptoms (LUTS), including benign prostatic obstruction (BPO). Eur J Urol.

[3] Silva J, Silva CM, Cruz F (2014). Current medical treatment of lower urinary tract symptoms/BPH: do we have a standard?. Curr Opin Urol.

[4] Oelke M (2013). EAU guidelines on the treatment and follow-up of non-neurogenic male lower urinary tract symptoms including benign prostatic obstruction. Eur Urol.

[5] Verhamme KM (2003). Treatment strategies, patterns of drug use and treatment discontinuation in men with LUTS suggestive of benign prostatic hyperplasia: the Triumph project. Eur Urol.

[6] Roehrborn CG (2008). Current medical therapies for men with lower urinary tract symptoms and benign prostatic hyperplasia: achievements and limitations. Rev Urol.

[7] Thomas JA, Tubaro A, Barber N (2016). A multicenter randomized noninferiority trial comparing GreenLght-XPS laser vaporiation of the prostate and transurethral resection of the prostate for the treatment of benign prostatic obstruction: 2 years outcomes of the COLIATH study. Eur J Urol.

[8] Rassweiler J (2006). Complications of transurethral resection of the prostate (TURP)-incidence, management, and prevention. Eur Urol.

[9] Cornu JN, Ayhai S, Bathmann A (2015). A systematic review and meta-analysis of functional outcomes and complications following transurethral procedures for lower urinary tract symptoms resulting from benign prostatic obstruction: an update. Eur J Urol.

[10] Magistro G, Stief C, Gratzke C (2015). New intraprostatic injectables and prostate urethral lift for male LUTS. Nat Rev Urol.

[11] Yu X (2008). Practice patterns in benign prostatic hyperplasia surgical therapy: the dramatic increase in minimally invasive technologies. J Urol.

[12] Füllhase C, Hakenberg O (2015). New concepts for the treatment of male lower urinary tract symptoms. Curr Opin Urol.

[13] Schauer I, Madersbacher S (2014). Medical treatment of lower urinary tract symptoms/benign prostatic hyperplasia: anything new in 2015. Curr Opin Urol.

[14] Barkin J (2012). UroLift system for relief of prostate obstruction under local anesthesia. Can J Urol.

[15] McNicholas TA (2013). Minimally invasive prostatic urethral lift: surgical technique and multinational experience. Eur Urol.

[16] Marlon P (2015). Prostatic urethral lift improves urinary symptoms and flow while preserving sexual function for men with benign prostatic hyperplasia: a systematic review and meta-analysis. Eur Urol.

[17] Roehrborn CG (2017). Five year results of the prospective randomized controlled prostatic urethral L.I.F.T. study. Can J Urol 2017.

[18] Sievert KD, Kunit T (2017). Emerging techniques in ‘truly’ minimal-invasive treatment options of bening prostatic obstruction. Curr Opin Urol.

[19] Gratzke C (2017). Prostatic urethral lift vs transurethral resection of the prostate: 2-year results of the BPH6 prospective, multicentre, randomized study. BJU Int.

[20] Woo HH (2011). Safety and feasibility of the prostatic urethral lift: a novel, minimally invasive treatment for lower urinary tract symptoms (LUTS) secondary to benign prostatic hyperplasia (BPH). BJU Int.

[21] Chin PT (2012). Prostatic urethral lift: two-year results after treatment for lower urinary tract symptoms secondary to benign prostatic hyperplasia. Urology.

[22] Roehrborn CG, Gange SN, Shore ND, Giddens JL, Bolton DM, Cowan BE, Cantwell AL, McVary KT, Te AE, Gholami SS, Moseley WG, Chin PT, Dowling WT, Freedman SJ, Incze PF, Coffield KS, Herron S, Rashid P, Rukstalis DB (2013). The prostatic urethral lift for the treatment of lower urinary tract symptoms associated with prostate enlargement due to benign prostatic hyperplasia: the L.I.F.T. Study. J Urol.

[23] Shore N (2014). Prospective multi-center study elucidating patient experience after prostatic urethral lift. Can J Urol.

[24] Sonkson J, Barber NJ, Speakman MJ (2015). Prospective, randomized multinational study of prostatic urethral lift versus transurethral resection of the prostate: 12 months results from the BPH6 study. Eur J Urol.

[25] Kranse R, van Mastrigt R (2003). Weak correlation between bladder outlet obstruction and probability to void to completion. Urology.

[26] Bruskewitz RC, Iversen P, Madsen PO (1982). Value of postvoid residual urine determination in evaluation of prostatism. Urology.

[27] Roehrborn CG (2016). Prostatic urethral lift: a unique minimally invasive surgical treatment of male lower urinary tract symptoms secondary to benign prostatic hyperplasia. Urol Clin N Am.

[28] McVary KT (2014). Treatment of LUTS secondary to BPH while preserving sexual function: randomized controlled study of prostatic urethral lift. J Sex Med.

[29] Berges R, Oelke M (2011). Age stratified normal values for prostate volume, PSA, maximum urinary flow rate, IPSS and other LUTS/BPH indicators in the German male community-dwelling population aged 50 years and older. World J Urol.

